# New-Onset Graves’ Disease Induced by COVID-19: A Case Report and Literature Review

**DOI:** 10.7759/cureus.73122

**Published:** 2024-11-06

**Authors:** Takashi Shinzato, Tadahiro Yonaha

**Affiliations:** 1 Department of General Internal Medicine, Nakagami Hospital, Okinawa, JPN

**Keywords:** covid-19, graves' disease, sars-cov-2, thyroid gland, thyrotoxicosis

## Abstract

We present a case of new-onset Graves' disease in a 40-year-old woman following COVID-19 infection. The patient experienced hand tremors, palpitations, shortness of breath with minimal exertion, and excessive sweating one week after recovering from COVID-19. Thyroid function tests revealed thyrotoxicosis, elevated free thyroxine, free triiodothyronine, and suppressed thyroid-stimulating hormone levels. Positive antithyroid peroxidase and thyroid-stimulating hormone (TSH) receptor antibodies, along with ultrasonography findings of diffuse thyroid enlargement and hypervascularization, confirmed the diagnosis of Graves' disease. Scintigraphy was omitted per the patient's request. This case contributes to the growing evidence suggesting SARS-CoV-2 may trigger autoimmune responses leading to thyroid disorders. We discuss the epidemiology, clinical characteristics, and potential mechanisms of Graves' disease following COVID-19, reviewing 28 similar cases reported from 2020 to early 2024. Our analysis reveals varied onset times and severity of thyroid dysfunction post-COVID-19, with some cases progressing to thyroid storm. Our findings highlight the importance of vigilant post-COVID-19 follow-up and contribute to understanding SARS-CoV-2's long-term consequences. From a cost-benefit perspective, a targeted screening approach might be needed for patients with persistent symptoms suggestive of thyroid dysfunction. This strategy could facilitate early detection and treatment, potentially preventing complications and reducing long-term healthcare costs.

## Introduction

Graves' disease is an autoimmune disorder characterized by hyperthyroidism, diffuse goiter, and ophthalmopathy [1]. It is caused by circulating antibodies that bind to and stimulate thyroid hormone receptors, resulting in hyperthyroidism and goiter. Several factors have been implicated in the pathogenesis, including viral infections, psychological stress, gender, smoking, thyroid damage, medications, and iodine exposure [2].

COVID-19, caused by severe acute respiratory syndrome coronavirus 2 (SARS-CoV-2), has led to significant morbidity and mortality worldwide. Recent studies suggest that COVID-19 might trigger autoimmune responses and lead to the onset or exacerbation of thyroid diseases, including Graves' disease, besides the primary respiratory manifestations [3]. We present a case of a 40-year-old woman who developed Graves' disease following COVID-19. This case and reviewing past case reports to identify the pattern of the onset and clinical characteristics contribute to understanding the potential association between COVID-19 and Graves' disease. They provide significant consideration into the potential effects of COVID-19 on thyroid function and the strategies of patient management and follow-up.

## Case presentation

A 40-year-old woman presented to our outpatient clinic with continuous hand tremors, palpitations, shortness of breath with minimal exertion, and excessive sweating. She had contracted COVID-19 one month prior, and her symptoms began one week after her fever subsided. She also reported alternating episodes of diarrhea. The patient had no past medical history and had never undergone thyroid function testing prior to this presentation, as she had been previously healthy with no symptoms suggestive of thyroid dysfunction. The patient had no known personal or family history of thyroid disease and took no regular medication. She was a lifelong nonsmoker and did not drink alcohol or use illicit drugs. She had received two vaccinations against SARS-CoV-2 in the two years preceding this presentation.

On examination at the first clinic visit, her temporal temperature was 37.1°C, heart rate 120 beats per minute with a regular rhythm, blood pressure 118/62 mmHg, respiratory rate 16 breaths per minute, and oxygen saturation 97% on ambient air. Fine tremors were observed on the outstretching of her hands, without sweaty palms. Diffuse thyroid enlargement was noted but was non-tender and without associated bruit. No exophthalmos was present. Cardiovascular, respiratory, and abdominal examinations were unremarkable.

Electrocardiography showed sinus tachycardia, and her chest radiograph was normal. A complete blood count examination showed slight leukopenia and normochromic normocytic anemia. Chemical investigations revealed normal renal function and moderately elevated liver enzymes, with aspartate aminotransferase 34 units/L, alanine aminotransferase 35 units/L, and gamma-glutamyltransferase 48 units/L. Total cholesterol was 120 mg/dL (Table 1).

**Table 1:** Results of extensive laboratory investigations at the presentation and at the time of follow-up after treatment following COVID-19 onset. TSH: thyroid-stimulating hormone; T4: thyroxine; T3: triiodothyronine *Treatment of methimazole started in the five weeks following COVID-19 onset. ^†^Measured using third generation assay.

Parameter	Reference range	Results on the week from COVID-19 onset*			
		4	8	15	19
White blood cells (/μL)	3,300-8,600	2,440	2,800	2,900	3,780
Hemoglobin (g/dL)	11.6-14.8	10.8	12.9	13.6	13.7
Hematocirt (%)	35.1-44.4	31.9	38.4	40.2	40.1
Platelets (/μL)	15.8-34.8	16.0 x10^4^	21.1 x10^4^	24.2 x10^4^	25.2 x10^4^
Aspartate transaminase (U/L)	13-30	34	22	15	15
Alanine transaminase (U/L)	7-23	35	24	11	11
Gamma-glutamyl transpeptidase (U/L)	9-32	48	65	37	26
Total cholesterol (mg/dL)	142-248	120	-	-	-
Urea (mg/dL)	8.0-20.0	11.7	11.5	9.2	9.2
Creatinine (mg/dL)	0.46-0.79	0.41	0.54	0.76	0.76
Sodium (mEq/L)	138-145	140	139	141	140
Potassium (mEq/L)	3.6-4.8	4.3	3.8	4.2	3.9
Erythrocyte sedimentation rate (mm/hr(	1-20	9	-	-	-
TSH (μUI/mL)	0.27-4.20	<0.002	<0.002	0.232	2.234
Free T4 (ng/dL)	0.9-1.8	6.87	1.03	0.94	0.98
Free T3 (pg/mL)	2.20-4.40	4.58	3.57	2.28	-
Anti-thyroglobulin antibodies (IU/mL)	<28.0	505.0	-	-	-
Anti-peroxidase antibodies (IU/mL)	<16.0	351.0	-	-	-
Anti-TSH receptor antibodies^†^ (IU/L)	<2.0	10.8	-	-	-

Thyroid function tests were suggestive of thyrotoxicosis, with free thyroxine (FT4) of 6.87 ng/dL (reference range: 0.9 to 1.8), free triiodothyronine of 4.58 pg/mL (reference range: 2.20 to 4.40), and thyroid-stimulating hormone (TSH) of <0.002 μU/mL (reference range: 0.27 to 4.0), respectively. Antithyroid peroxidase antibody and TSH receptor antibody (TRAb) (using third-generation assay) were both positive, with titers of 351.0 IU/mL (reference range: <16.0) and 10.8 IU/L (reference range: <2.0) (Table 1).

**Figure 1:**
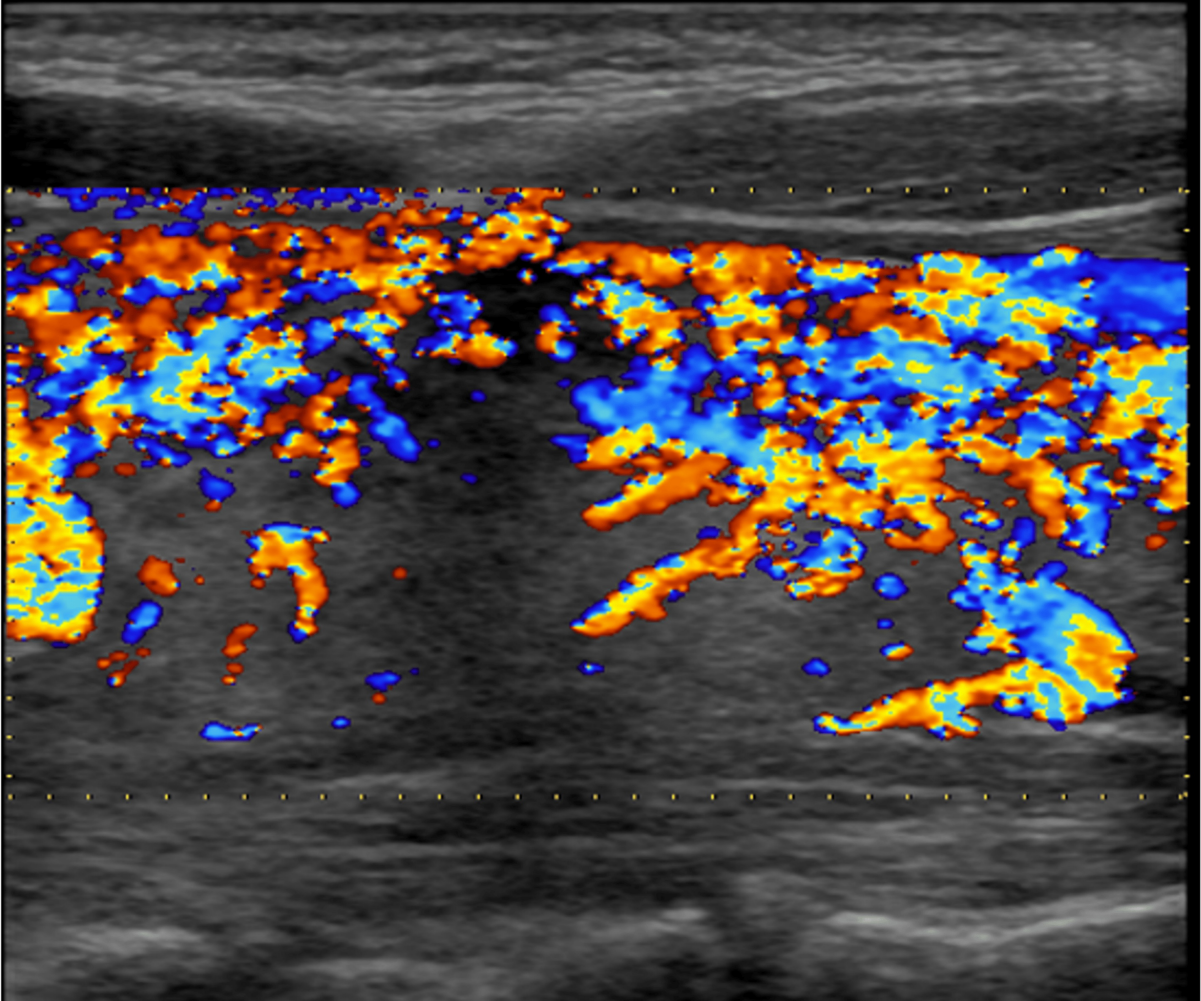
The features of the right thyroid lobe on color Doppler mode. The flow of the right lobe increased, as also shown in the left lobe.

Thyroid ultrasonography showed diffuse thyroid enlargement of both lobes with isthmus (grade 2 goiter) and heterogeneous hypoechoic parenchyma. Both lobes were hypervascular, more evident on the right. The color Doppler mode revealed a significant increase in blood flow intensity (Figure 1). Her clinical presentation and these findings were consistent with Graves' disease. She had expressed a desire for future pregnancy, so scintigraphy was not performed.

The patient was started on antithyroid medication (methimazole, 30 mg/day) to control thyrotoxicosis. After three weeks of treatment, her clinical condition improved. Her blood counts, liver enzymes, and FT4 reached normal levels. Methimazole was titrated every four weeks to a low-maintenance dose of 2.5 mg. After three months of treatment, an euthyroid state was achieved (Table 1).

## Discussion

The diagnosis of Graves' disease in the present case was based on the patient's history, physical examination, blood test results, and thyroid ultrasound findings, aligning with the 2016 American Thyroid Association guidelines for diagnosis and management of hyperthyroidism and other causes of thyrotoxicosis [4]. The positive third-generation TRAb test further supported the diagnosis, with its high sensitivity (97%) and specificity (99%) for Graves' disease [5]. The patient had no prior thyroid disease, and it also indicated that SARS-CoV-2 could potentially trigger new-onset Graves' disease in previously healthy individuals. There is the possibility of pre-existing subclinical disease, the temporal relationship between COVID-19 and the acute onset of symptoms strongly suggests a causal relationship.

Recent studies suggest that the mechanisms by which COVID-19 induces Graves' disease may differ from those associated with other viral infections. SARS-CoV-2 has been shown to play a unique role in the pathogenesis of hyperthyroidism in Graves' disease. Unlike other viruses that may indirectly affect thyroid function through immune system activation, SARS-CoV-2 appears to be able to interact with the thyroid gland directly [6,7]. SARS-CoV-2 might induce hyperthyroidism by binding to angiotensin-converting enzyme II receptors, which are highly expressed in the thyroid gland, leading to the increase of serum levels of 3,5,30-triiodothyronine and thyroxine [7]. In addition, the systemic inflammatory response triggered by COVID-19 characterized by a cytokine storm may exacerbate the autoimmune response, further promoting the onset of Graves' disease [6,7]. The mechanism contrasts with other viral infections, where thyroid involvement is often secondary to immune system dysregulation rather than direct viral action.

We reviewed the characteristics of 28 patients with SARS-CoV-2-induced new onset or relapse of Graves' disease reported from 2020 through early 2024, including the present one, revealing several clinical patterns (Table 2) [8-28]. The patients ranged in age from 16 to 81, with a mean age of 40. Most cases were reported in middle-aged adults like our patient. The female is predominant, constituting 23 cases (85%), and it aligns with the known epidemiology of Graves' disease [1].

**Table 2:** Clinical characteristics of 28 cases of new onset or relapse of Graves' disease following COVID-19 in the literature. F: female; M: male; NA: not available

Author, year of publication [reference]	Age (year)	Gender	Past thyroid history	Family thyroid history	Type of onset	Time to presentation following COVID-19	Status on presentation	Setting for treatment
Pastor et al. 2020 [8]	45	F	Graves' disease	NA	Relapse	Concomitant	Thyroid storm	Hospitalization, corticosteroids required
Mateu-Salat et al. 2020 [9]	60	F	Graves' disease	NA	Relapse	1 month	Thyroid toxicity	Outpatient clinic
Mateu-Salat et al. 2020 [9]	53	F	No	NA	New onset	2 months	Thyroid toxicity	Outpatient clinic
Jiménez-Blanco et al. 2021 [10]	45	F	Graves' disease	NA	Relapse	Concomitant	Thyroid toxicity	Outpatient clinic
Jiménez-Blanco et al. 2021 [10]	61	F	Graves' disease	NA	Relapse	1 month	Thyroid toxicity	Hospitalization
Harris and Al Mushref 2021 [11]	21	F	No	Hypothyroidism (mother)	New onset	16 days	Thyroid toxicity	Outpatient clinic
Lanzolla et al. 2021 [12]	33	F	No	NA	New onset	2 months	Thyroid toxicity	Outpatient clinic
Edwards and Hussain 2021 [13]	27	M	No	No	New onset	Concomitant	Thyroid storm	Hospitalization, corticosteroids required
Edwards and Hussain 2021 [13]	21	F	No	No	New onset	Concomitant	Impending storm	Hospitalization, corticosteroids required
Montebello 2021 [14]	22	F	Graves' disease	NA	Relapse	2 months	Thyroid toxicity	Outpatient clinic
Feghali et al. 2021 [15]	33	F	No	NA	New onset	7 weeks	Thyroid toxicity	Outpatient clinic
Milani et al. 2021 [16]	39	F	Graves' disease	NA	Relapse	2 weeks	Thyroid storm	Hospitalization, corticosteroids required
Milani et al. 2021 [16]	50	M	Hyperthyroidism	NA	Relapse	Concomitant	Thyroid storm	Hospitalization, corticosteroids required
Urbanovych et al. 2021 [17]	22	F	No	NA	New onset	3 weeks	Impending storm	Outpatient clinic, corticosteroids required
Rockett et al. 2021 [18]	16	M	No	NA	New onset	19 days	Impending storm	Hospitalization
Mohammed et al. 2021 [19]	28	F	Graves' disease	NA	Relapse	28 days	Thyroid toxicity	Outpatient clinic
Pranasakti et al. 2022 [20]	26	F	No	NA	New onset	Concomitant	Thyroid storm	Hospitalization, NA for corticosteroids
Ghareebian and Mariash 2022 [21]	48	M	No	NA	New onset	Concomitant	Impending storm	Hospitalization, corticosteroids required
França et al. 2023 [22]	30	F	No	No	New onset	3 months	Thyroid toxicity	Outpatient clinic
Boyle and Mullally 2023 [23]	65	F	No	NA	New onset	2 weeks	Impending storm	Hospitalization
Nham et al. 2023 [24]	27	F	No	NA	New onset	2 weeks	Thyroid toxicity and concurrent subacute thyroiditis	Outpatient clinic
Shermetaro and Bushman 2023 [25]	81	M	No	No	New onset	1 week	Thyroid storm	Hospitalization, corticosteroids required
Sebastian et al. 2024 [26]	42	F	Graves' disease	NA	Relapse	3 weeks	Impending storm	Outpatient clinic
Du et al. 2024 [27]	53	F	Hashimoto’s thyroiditis	NA	New onset	1 month	Thyroid toxicity	Outpatient clinic
Du et al. 2024 [27]	30	F	Hashimoto’s thyroiditis	NA	New onset	1 month	Thyroid toxicity	Outpatient clinic
Du et al. 2024 [27]	32	F	Graves' disease	NA	Relapse	2 months	Thyroid toxicity	Outpatient clinic
Deng et al. 2024 [28]	60	F	No	NA	New onset	10 days	Thyroid with liver and kidney injury toxicity	Hospitalization

These cases suggest that SARS-CoV-2 can act as a trigger for both relapses in predisposed individuals and new-onset in healthy ones. Family history was not commonly reported, but where available, it indicated a genetic predisposition, such as hypothyroidism in a mother. The time to presentation following COVID-19 infection varied widely, from as early as concomitant or within seven days to more than two months.

The severity of thyroid dysfunction at presentation varied among the cases reviewed. Twelve patients developed an impending storm or thyroid storm, requiring hospitalization and aggressive treatment, including corticosteroids [8,10,13,16,18,20,21,23,25,27]. The remaining 16 patients had thyroid toxicity managed in outpatient settings. Our patient, who presented with significant thyroid toxicity but did not require hospitalization, underscores the importance of early detection and management of thyroid dysfunction in post-COVID-19 patients. Clinicians should consider these findings for thyroid dysfunction in the context of post-COVID-19 care, even when faced with the challenges of distinguishing these symptoms from the myriad of post-COVID complications.

## Conclusions

The presentation of Graves' disease in our patient, including hand tremors, palpitations, and shortness of breath, aligns with other reported cases of post-COVID-19 Graves' disease. Many patients experience the onset of thyroid dysfunction within weeks of recovering from the acute phase of COVID-19. Laboratory findings typically show suppressed TSH levels, elevated free triiodothyronine and FT4 levels, and positive thyroid autoantibodies. From a cost-benefit perspective, routine thyroid function screening for all post-COVID-19 patients might not be economically feasible or medically necessary. However, for patients presenting with persistent symptoms suggestive of thyroid dysfunction, the benefits of early detection and treatment likely outweigh the screening costs. This approach might allow for timely intervention before developing more severe complications such as a thyroid storm.
